# *Cyathea gigantea* (Cyatheaceae) as an antimicrobial agent against multidrug resistant organisms

**DOI:** 10.1186/s12906-019-2696-0

**Published:** 2019-10-22

**Authors:** Kathakali Nath, Anupam Das Talukdar, Mrinal Kanti Bhattacharya, Deepshikha Bhowmik, Shiela Chetri, Debarati Choudhury, Abhijit Mitra, Nargis Alom Choudhury

**Affiliations:** 1 Department of Microbiology, Assam University, Silchar, India; 2 Department of Life Science and Bio informatics, Assam University, Silchar, India; 3 Department of Botany and Biotechnology, Karimganj College, Karimganj, India

**Keywords:** *Cyathea gigantea*, Cyatheaceae, Extracts, Antibiotics, MDR bacteria, Antibacterial, Synergy

## Abstract

**Background:**

Rapid emergence of multidrug resistant (MDR) organisms in hospital and community settings often result into treatment failure, thus leading the clinicians with fewer treatment options. *Cyathea gigantea*, an ethnomedicinally important fern used in cuts and wound infections. So, if this medicinal plant is used in treating the MDR infections then it might bring certain relief in future treatment options.

**Methods:**

Antibacterial activity of *C. gigantea* against MDR bacteria was assed using well diffusion and broth microdilution methods to determine the diameters of growth inhibition zones, minimum inhibitory concentration (MIC) and minimum bactericidal concentration (MBC). Synergistic activity was also determined with the conventional antibiotics by disc diffusion method followed by FIC index of each of the tested antibiotic was calculated. The active extract was then subjected to fractionation by column chromatography and antibacterial activity was done with each of the collected fractions.

**Results:**

Crude extract of *C. gigantea* was found to be active against all the tested organisms. The MIC was 200 μg/ml against Gram-positive i.e., *Staphylococcus aureus* ATCC 25923 and 400 μg/ml against Gram-negative i.e., *Escherichia coli* ATCC 25922 and *Pseudomonas aeruginosa* PAO1, while the MBC was 400 μg/ml in case of Gram-positive and 800 μg/ml for Gram-negative. The synergistic activity revealed that the plant extract increased the antibacterial property of the studied antibiotics and the FIC index showed that significant synergistic activity was shown by ciprofloxacin followed by tetracycline, ampicillin and oxacillin. Antibacterial activity with the fractionated extract showed that the FR II, FR III and FR IV were active against both Gram-positive and Gram-negative bacteria, whereas FR I, FR V and FR VI did not show antibacterial property against any of the tested bacteria.

**Conclusions:**

Extracts of *C. gigantea* was found active against both selected Gram-positive and Gram-negative organisms and thus offers the scientific basis for the traditional use of the fern. The present study also provides the basis for future study to validate the possible use against multidrug resistant organisms.

## Background

The worldwide emergence of multidrug resistant bacteria is the primary cause of various treatment failure and thus leading the clinicians with fewer treatment options. Natural products of plant origin are sources of various secondary metabolites and thus known to possess potential antimicrobial properties. In particular, the drugs derived from natural products are important in the treatment of life-threatening conditions [1]. Hence, if medicinal plants are used in treating the MDR infections, they might bring certain relief in future treatment options.

The fern possesses an important role in folklore medicine although neglected in modern days. The family Cyatheaceae includes the worlds’ tallest tree ferns. *C. gigantea* is an ethno-medicinal important fern and a native of Southern Assam [2].Paste of apical soft portion of caudex is applied on cuts and wounds which prevents microbial growth and inhibits abscess formation. The plant is also effective in immediate blood clotting [3–5]. Hentriacontane, âsitostanone, diploterol, sitosterol, hop-29-ol and oleanolic acid were isolated from the hexane-soluble fraction of the tree fern, *C. gigantea* [6]. The phytochemical study showed the presence of steroid, flavonoid and saponin and TLC profiling of plant extracts in different solvent system confirms the presence of diverse group of phytochemicals [7].

Therefore, in this study an attempt has been taken to explore the antibacterial property of the fern *C. gigantea* available in Southern part of Assam, India.

## Methods

### Plant material

Fresh plant material (fronds) of *C. gigantea* was collected from Southern Assam. The identification of the fern was done by Miss Kathakali Nath using standard manual [8, 9] and was formally confirmed by Dr. M. K. Bhattacharya. It was also matched with the available herbarium of the species at Central National Herbarium of Botanical Survey of India, Calcutta. A voucher specimen of the fern has been deposited in the Karimganj College Herbarium, Karimganj, Assam bearing deposition number M. 1213.

### Extraction and fractionation

With tap and subsequently by sterile water the fresh fronds were cleaned, which were then shad-dried and pounded to powder. For extraction 50 g of powdered plant material was added to three solvents (500 ml) of increasing polarity (ethyl acetate, methanol and water) using soxlet evaporator [10]. Boiling method was adopted for preparation of water extract.

The ethyl acetate extract (most active extract) was fractionated by using silica gel coloumn chromatography with different ratios of solvents i.e., petroleum ether, ethyl acetate and methanol and the fractions were named as FR I (only petroleum ether), FR II (PE-90:EA-10), FR III (PE-85:EA-10:MeOH-5), FR IV (PE-80:EA-20), FR V (PE-80:EA-10:MeOH-10) and FR VI (PE-75:EA-25). The collected fractions were then evaluated for their antibacterial activity [11].

### Micro-organisms

The strains (KKN) were isolated from Silchar Medical College and Hospital Silchar, India from the patients who were admitted or visited the clinics of the hospital. The samples were collected on recommendation of the clinician for routine culture and sensitivity in the Department of Microbiology of Silchar Medical College and Hospital. The investigators have collected the isolates from the above mentioned Department and characterized for ESBL producing *E. coli* (KKN5 and KKN 6) and MBL producing *P. aeruginosa* (KKN1 and KKN2) production using PCR based assay and sequencing. The same was done for characterization of MRSA (KKN3, KKN4, KKN10, KKN11, and KKN12) strains as well. *E. coli* ATCC 25922 and *S. aureus* ATCC 25923 and *P. aeruginosa* PAO1 were used as controls.

### Antibacterial activity of extracts and fractions from *C. gigantea*

#### Determination of diameters of growth inhibition zones

Agar well diffusion method was used for determining the antibacterial activity of different extracts. Aided with sterile swab, each bacterial culture (10^5^ cfu/ml) was spread on Mueller Hinton Agar plates. Followed by wells (5 mm diameter) were punched in the agar medium where 20 μl of extract was added along which same volume of dimethyl sulphoxide (DMSO) served as negative control and antibiotic discs of ampicillin (10 μg) and ciprofloxacin (5 μg) were used as positive control. The plates were then incubated and the zone of inhibition was measured in mm [12]. The test was carried out in triplicate.

The different fractions obtained were allowed to stand at room temperature till the solvent evaporated. The dried fractions were dissolved in di-methyl sulphoxide (DMSO) and used for screening the antibacterial activity against the reference strains of *E. coli* ATCC 25922, *P. aeruginosa* PAO1 and *S. aureus* ATCC 25923 by well diffusion method [11].

#### Determination of minimum inhibitory concentrations (MIC) and determination of minimum bactericidal concentrations (MBC)

Broth dilution method was performed for determination of MIC. Test tubes containing culture media along with different dilutions of extract i.e., 25–800 μg/ml dissolved in dimethyl sulphoxide (DMSO) was used. To each of the test tubes bacterial cultures (10^5^ cfu/ml) were added. Positive control containing growth media with each organisms and negative control containing only extract and media was used [13]. The test was carried out in triplicate.

For determination of MBC, test tubes from MIC study where no visible growth observed were subsequently sub-cultured (100 μl) into agar medium [14]. The test was carried out in triplicate.

#### Association of extracts with conventional antibiotics

Bacterial culture of 10^5^ cfu/ml were seeded on Mueller Hinton agar plates to which antibiotic disc viz. ampicillin (2 μg), ciprofloxacin (5 μg), erythromycin (15 μg), oxacillin (1 μg) and tetracyclin (30 μg) were placed. To each of the disc 6 μl of extract (1x MIC) was added. The plates were then incubated [15] and the results were interpreted as per CLSI guidelines 2015 [16]. The test was carried out in triplicate.

The FIC index of each antibiotic combined with extract was calculated using eq. 3.

1 $$ \mathrm{FIC}\ \mathrm{A}\ \left(\mathrm{extract}\right)=\mathrm{MIC}\ \mathrm{of}\ \mathrm{extract}\ \mathrm{in}\ \mathrm{combination}/\mathrm{MIC}\ \mathrm{of}\ \mathrm{extract}\ \mathrm{alone} $$

2 $$ \mathrm{FIC}\ \mathrm{B}\ \left(\mathrm{antibiotic}\right)=\mathrm{MIC}\ \mathrm{of}\ \mathrm{antibiotic}\ \mathrm{in}\ \mathrm{combination}/\mathrm{MIC}\ \mathrm{of}\ \mathrm{antibiotic}\ \mathrm{alone} $$

The sum of FIC A and FIC B determines FIC index (eq. 3)

3 $$ \mathrm{FIC}\ \mathrm{index}=\mathrm{FIC}\ \mathrm{A}+\mathrm{FIC}\ \mathrm{B} $$

The effects of combination results into synergy, indifference, antagonism and additive and is defined as ∑FIC ≤ 0.5, ∑FIC ≤ 4, ∑FIC > 4 and 0.5 ∑FIC ≤ 1 respectively

#### Gas chromatography mass spectroscopy

Gas chromatography mass spectroscopy was done at AIRF, Jawaharlal Nehru University, Delhi and was carried out with GCMS-QP2010 Plus, Shimadzu, Kyoto, Japan. The type of column used was RXi-5 Sil MS (30 m X 0.25 mm i.d. X 0.25 μm film thickness) whose initial temperature was 60 °C and the carrier gas used was helium. The compounds were identified using two libraries i.e., NIST14.lib and WILEY8.lib (Additional file 1).

#### Statistical analysis

All the values were expressed as mean ± standard deviation of three replicates. Statistical significance was determined by *p*-value < 0.05.

## Results

The ethyl acetate, methanol and water extracts of *C. gigantea* were studied for their antibacterial activities against MDR bacteria. Of the three extracts, ethyl acetate extract was found to possess antibacterial activity especially against *E. coli, P. aeruginosa, S. aureus* as well as against reference strains, *E. coli* ATCC 25922 and *S. aureus* ATCC 25923. The diameters of inhibition zones varied from 11 to 13 mm and from 8 to 12 mm against Gram-negative and Gram-positive bacteria, respectively (Table 1).

**Table 1:** Diameters of inhibition zones (mm) of the ethyl acetate extract of *C. gigantea*

	*Escherichia coli*			*Pseudomonas aeruginosa*			*Staphylococcus aureus*					
	ATCC 25922	KKN 5	KKN 6	PAO 1	KKN 1	KKN 2	ATCC 25923	KKN 3	KKN 4	KKN10	KKN11	KKN12
Ethyl acetate extract	10.67 ± 0.577	12.33 ± 0.577	11.17 ± 0.288	11.33 ± 0.577	12.67 ± 0.577	13.83 ± 0.763	11.5 ± 1.32	10.5 ± 0.500	12.67 ± 0.577	11.33 ± 1.52	9.67 ± 1.52	10.67 ± 0.763

The test was done in triplicate. Diameter of the zone of inhibitions (mm) is given here as mean ± standard deviation

Since the ethyl acetate extract of *C. gigantea* was found to possess significant antibacterial property so, the ethyl acetate extract was fractionated by column chromatography. The elution was done using petroleum ether, ethyl acetate and methanol in different ratios and six fractions were collected. A varying degree of antibacterial activity was observed with the FR II, FR III and FR IV fractions of ethyl acetate extract against all the tested organisms. Highest zone of inhibition was shown by FR IV fraction followed by FR II and FR III fraction in case of Gram-positive i.e., *S. aureus*, whereas in case of Gram-negative a consistent increase was observed from FR II to FR IV which was in accordance with increase in polarity of the solvent. However, FR I, FR V and FR VI did not show any antibacterial property.

The MIC assay was carried with ethyl acetate extract at25μg/ml, 50 μg/ml, 100 μg/ml, 200 μg/ml, and 400 μg/ml concentration. 200 μg/ml was found to be the MIC against *S. aureus* ATCC 25923 and 400 μg/ml was the MIC against *E. coli* ATCC 25922 and *P. aeruginosa* PAO1 (Table 2).

**Table 2:** Minimum inhibitory concentration (MIC) and Minimum bactericidal concentration (MBC)

Organisms		Concentrations (μg/ml) of ethyl acetate crude extract
*Staphylococcus aureus* ATCC 25923	MIC	200 ± 0
	MBC	400 ± 0
*Escherichia coli* ATCC 25922	MIC	400 ± 0
	MBC	800 ± 0
*Pseudomonas aeruginosa* PAO1	MIC	400 ± 0
	MBC	800 ± 0

The MBC values (Table 3) were found to be two-fold greater than the MIC values on the corresponding microorganisms.

**Table 3:** FIC index of the tested antibiotic

FIC index				
Ampicillin	Ciprofloxacin	Erythromycin	Oxacillin	Tetracycline
1.125 ± 0.11	0.65 ± 0.10	0.96 ± 0.11	1.75 ± 0	0.96 ± 0.10
0.812 ± 0.09	0.57 ± 0.08	0.73 ± 0.09	1.12 ± 0.11	0.73 ± 0.22
0.656 ± 0.12	0.57 ± 0.23	0.73 ± 0	1.12 ± 0	0.73 ± 0.12
0.812 ± 0.13	0.57 ± 0.12	0.73 ± 0	1.12 ± 0.11	0.73 ± 0.20
0.656 ± 0.11	0.57 ± 0.22	0.73 ± 0.21	1.12 ± 0	0.73 ± 0.09
0.812 ± 0.11	0.57 ± 0.15	0.61 ± 0	0.81 ± 0	0.73 ± 0

**Fig. 1:**
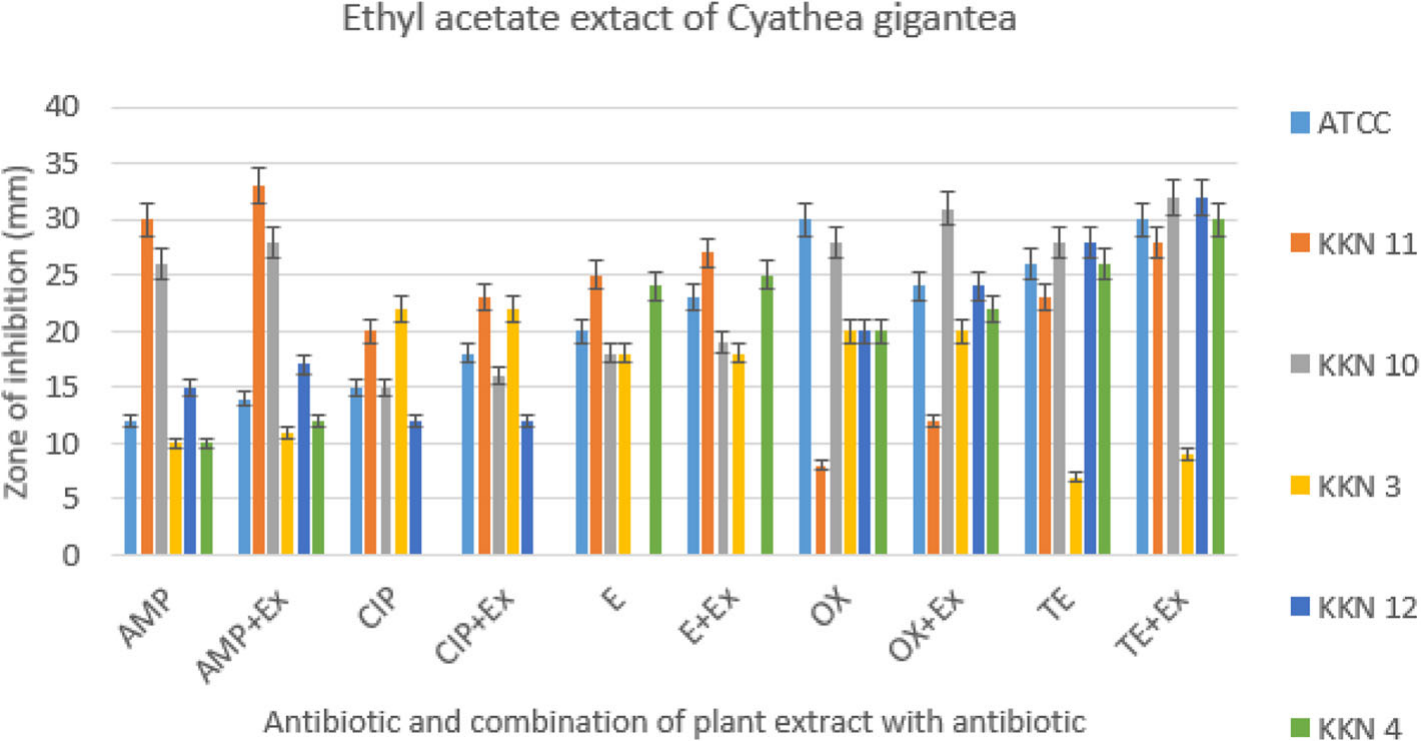
Synergistic activity with the ethyl acetate crude extract against methicillin resistant *Staphylococcus aureus*

The combination of the ethyl acetate extract of *C. gigantea* with conventional antibiotics: ampicillin, ciprofloxacin, erythromycin, oxacillin and tetracycline against methicillin resistant *S. aureus* (Fig. 1) and reference strain, *S. aureus* ATCC 25923, revealed that the antibacterial activity of all studied antibiotics increased in the presence of the plant extract. The FIC index of each antibiotic combined with extract was calculated using eq. 3 (Table 3). The analysis of results revealed synergistic effects with ciprofloxacin, tetracycline, ampicillin and oxacillin (Table 4).

**Table 4:** Diameters of inhibition zones (mm) of fractions from *C. gigantea* ethyl acetate extract

	FR II			FR III			FR IV		
	100	200	400	100	200	400	100	200	400
*Staphylococcus aureus* ATCC 25923	15.33 ± .577	–	–	10.5 ± 0.5	–	–	23.17 ± .76	–	–
KKN 3	12 ± 1.5	–	–	1.33 ± 1.04	–	–	21.5 ± 1.32	–	–
KKN 4	16.5 ± 0.5	–	–	8.67 ± 1.15	–	–	21.83 ± 1.89	–	–
KKN 10	11.33 ± .288	–	–	9.167 ± 1.75	–	–	22.33 ± 1.52	–	–
KKN 11	13.33 ± .288	–	–	11.33 ± 1.52	–	–	23.47 ± .503	–	–
KKN 12	12.167 ± 1.755	–	–	10.33 ± .289	–	–	20.33 ± .577	–	–
*Escherichia coli* ATCC 25922	–	–	7.167 ± .751	–	–	15.167 ± 1.25	–	–	24.5 ± 0.5
KKN 5	–	–	6.67 ± 1.154	–	–	13.13 ± 1.20	–	–	23.5 ± .866
KKN 6	–	–	6.83 ± 1.44	–	–	12 ± 0.5	–	–	22.467 ± 1.36
*Pseudomonas aeruginosa* PAO1	–	–	8.67 ± .763	–	–	11.33 ± 1.52	–	–	26.067 ± .901
KKN 1	–	–	9.33 ± .288	–	–	9.67 ± .577	–	–	23.83 ± .763
KKN 2	–	–	7.67 ± .577	–	–	9.5 ± 0.5	–	–	23.83 ± 1.44

The test was done in triplicate. Diameter of the zone of inhibitions is given here as mean ± standard deviation

The GC-MS analysis of the three fractions i.e., FR II, FR III and FR IV of *Cyathea gigantea* revealed the presence of 64 phytocomponents in total. Of which, on literature survey 11 phyto constituents i.e., Isophytol; N-Hexadecanoic acid; Phytol; N-Tetracosanol-1; 1-heptacosanol; Octacosanol; Hexadecanoic acid, 2-hydroxy-1- (hydroxymethyl)ethyl ester; Octadecanoic acid; 1-eicosanol Cycloheptasiloxane, tetradecamethyl- and Neophytadiene (Table 5) were reported to possess antimicrobial property.

**Table 5:** Phytocomponents identified in the three fractions of ethyl acetate extract of *Cyathea gigantea* possessing antimicrobial property

Sl no.	Fractions	Name of compound	Molecular weight	Retention time	Peak area	Bioactivity
FR II						
1.		Isophytol	296.539 g/mol	23.614	599,052	Antimicrobial [17]
2.		N-Hexadecanoic acid	256.430 g/mol	23.930	19,929,360	Anti-inflammatory, Antioxidant, nematicide, pesticide [18]
3.		Phytol	296.539 g/mol	25.858	3,898,743	Antioxidant activities, anti-inflammatory and antiallergic effects, antimicrobial activity [19]
4.		N-Tetracosanol-1	354.663 g/mol	26.949	6,510,251	Anti-bacterial activity [20]
5.		1-heptacosanol	396.744 g/mol	29.384	3,641,493	Nematicidal, anticancer, antioxidant and antimicrobial [21]
6.		Octacosanol	410.771 g/mol	35.391	1,016,504	Antinociceptive and anti-inflammatory [22]
7.		Cycloheptasiloxane, tetradecamethyl-	519.078 g/mol	15.954	17,051,468	Skin-Conditioning Agent, Fragrance, antimicrobial [23]
8.		Neophytadiene	278.524 g/mol	22.028	3,619,028	antipyretic, analgesic, and anti-inflammatory, antimicrobial, antioxidant [21]
FR III						
9.		N-Hexadecanoic acid	256.430 g/mol	23.835	1,135,466	Anti-inflammatory, Antioxidant, nematicide, pesticide [18]
10.		N-Tetracosanol-1	354.663 g/mol	26.947	6,089,033	Anti-bacterial activity [21]
11.		1-heptacosanol	396.744 g/mol	29.381	3,440,611	Nematicidal, anticancer, antioxidant and antimicrobial [21]
12.		Hexadecanoic acid, 2-hydroxy-1-(hydroxymethyl)ethyl ester	330.509 g/mol	30.533	715,796	Pesticide, antioxidant [24]
FR IV						
13.		N-Hexadecanoic acid	256.430 g/mol	23.847	8,140,130	Anti-inflammatory, Antioxidant, nematicide, pesticide [18]
14.		Octadecanoic acid	284.484 g/mol	26.537	1,596,370	Not reported
15.		N-Tetracosanol-1	354.663 g/mol	26.953	6,793,576	Anti-bacterial activity [21]
16.		1-heptacosanol	396.744 g/mol	33.022	1,751,095	Nematicidal, anticancer, antioxidant and antimicrobial [21]
17.		1-eicosanol	298.555 g/mol	35.398	749,812	Antimalarial, antifungal, antioxidant [25]

## Discussion

The spread of drug-resistant microorganisms is a serious threat to successful therapy of microbial diseases. Therefore there is an urgent need to search new compounds characterized by diverse chemical structures and mechanisms of action. Plants are rich in a wide variety of secondary metabolites such as tannins, terpenoids, alkaloids and flavonoids which have been found in vitro to possess antimicrobial properties.

*C. gigantea*, a tree fern belonging to the family Cyatheaceae, possesses immense and diverse ethnomedicinal importance [3–5]. Preliminary phytochemical analysis of different extract of the fern was carried out which have brought foreword the presence of various bioactive secondary metabolites viz., steroids, alkaloids, phenolic groups, cardiac glycosides, flavonoids, saponins, tannins and terpenoids [26]. This presence of bioactive secondary metabolites has investigated to study the plant for its various bioactivity. In this study the antibacterial activity of *C. gigantea* is evaluated. Ethyl acetate extract was recorded to be the active extract against all the tested organisms and this might be due to the presence of various phytochemicals. The antimicrobial property also may be due to the presence of coumarin, which is an antimicrobial agent naturally present in plant [27]. In addition some other isolated compounds viz., 2-methylbutane-1,4-diol, 3-(1-ethoxyethoxy) may also impart to the antibacterial property of the plant [26].

The fractionated crude ethyl acetate extract also showed potential antibacterial activity against both Gram-positive and Gram-negative. Of the six fractions, FR II, FR III and FR IV exhibited antibacterial property while FR I, FR V and FR VI did not show any activity. The fractions (FR II, FR III and FR IV) showed significant variations in their inhibition zones against the studied organisms.

The MIC was found to be 200 μg/ml against Gram-positive and 400 μg/ml against Gram-negative bacteria. The efficacy of the extract is determined by its MIC value. Extracts having MIC value lesser than 1000 μg of crude extracts or 100 μg of isolated compounds is considered to be effective concentration. In the study the MIC value varied from 200 to 400 μg/ml against the tested bacteria indicating the fern to be an effective antibacterial agent. The higher MIC against Gram-negative i.e., *E. coli* ATCC 25922 and *P. aeruginosa* PAO1 in comparison to *S. aureus* ATCC 25923 may be due to the presence of outer membrane in their cell wall [28].

The differences in MBC and MIC suggests that the extract possesses a selective antibacterial property. The extract was found to show good bactericidal activity against the tested organisms as the ratio of MBC/MIC was equals to 2 [29].

The present study also focused on the combined effect of ethyl acetate extract and antibiotics. The results revealed the synergistic and additive interactions between the phytochemicals present in the ethyl acetate extract and antibiotics, which are cell wall inhibitors (ampicillin, oxacillin), protein synthesis inhibitor (erythromycin, tetracycline) and DNA synthesis inhibitor (ciprofloxacin). The FIC index showed that significant synergistic activity was shown by ciprofloxacin followed by tetracycline, ampicillin and oxacillin.

## Conclusion

Extracts of *C. gigantea* showed activity against both Gram-positive and Gram-negative organisms and thus offers the scientific basis for the traditional use of the fern. The study also highlighted that FR IV of ethyl acetate extract of *C. gigantea* showed the highest antibacterial activity which is due to the presence of various active phytochemicals with high peak area shown in GCMS. The present study also provides the basis for future study to validate the possible use against multidrug resistant organisms.

## Supplementary information

**Additional file 1.** Identifying High Risk Areas of Zika Virus Infection by Meteorological Factors in Colombia

## Data Availability

The datasets used and/or analysed during the current study available from the corresponding author on reasonable request.

## Abbreviations

CLSI: Clinical Laboratory Standard Institute
DMSO: Dimethyl Sulphoxide
EA: Ethyl Acetate
ESBL: Extended Spectrum Beta Lactamase
FIC: Fractional Inhibitory Concentration
GCMS: Gas Chromatography Mass Spectroscopy
MBC: Minimum Bactericidal Concentration
MBL: Metallo Beta Lactamase
MDR: Multi Drug Resistant
MeOH: Methanol
MIC: Minimum Inhibitory Concentration
MRSA: Methicillin Resistant *Staphylococcus aureus*
PCR: Polymerase Chain Reaction
PE: Petroleum Ether
TLC: Thin Layer Chromatography
