# Supplementary material for: Cyathea gigantea (Cyatheaceae) as an antimicrobial agent against multidrug resistant organisms
Source: BMC Complement Altern Med. 2019 Oct 22;19:279. doi: 10.1186/s12906-019-2696-0 (PMC6805519; doi:10.1186/s12906-019-2696-0)
Supplement: Supplementary file 1 — Additional file 1. Identifying High Risk Areas of Zika Virus Infection by Meteorological Factors in Colombia [file 12906_2019_2696_MOESM1_ESM.pdf]

D:\GCMS\GCMS METHOD\Organic\Extract.qgm

Method

[Comment]

===== Analytical Line 1 =====

[AOC-20i+s]

# of Rinses with Presolvent	:5
# of Rinses with Solvent(post)	:8
# of Rinses with Sample	:2
Plunger Speed(Suction)	:High
Viscosity Comp. Time	:0.2 sec
Plunger Speed(Injection)	:High
Syringe Insertion Speed	:High
Injection Mode	:Normal
Pumping Times	:5
Inj. Port Dwell Time	:0.0 sec
Terminal Air Gap	:No
Plunger Washing Speed	:High
Washing Volume	:6uL
Syringe Suction Position	:0.0 mm
Syringe Injection Position	:0.0 mm
Solvent Selection	:All A,B,C

[GC-2010]

Column Oven Temp.	:60.0 °C
Injection Temp.	:260.00 °C
Injection Mode	:Split
Flow Control Mode	:Linear Velocity
Pressure	:73.2 kPa
Total Flow	:16.3 mL/min
Column Flow	:1.21 mL/min
Linear Velocity	:40.1 cm/sec
Purge Flow	:3.0 mL/min
Split Ratio	:10.0
High Pressure Injection	:OFF
Carrier Gas Saver	:OFF
Splitter Hold	:OFF

Oven Temp. Program

Rate	Temperature(°C)	Hold Time(min)
-	60.0	2.00
7.00	250.0	0.00
15.00	280.0	29.00

< Ready Check Heat Unit >

Column Oven	: Yes
SPL1	: Yes
MS	: Yes

< Ready Check Detector(FTD) >
< Ready Check Baseline Drift >
< Ready Check Injection Flow >

SPL1 Carrier	: Yes
SPL1 Purge	: Yes

< Ready Check APC Flow >
< Ready Check Detector APC Flow >

External Wait	:No
Equilibrium Time	:1.0 min

[GC Program]

[GCMS-QP2010 Ultra]

IonSourceTemp	:230.00 °C
Interface Temp.	:270.00 °C
Solvent Cut Time	:4.50 min
Detector Gain Mode	:Relative
Detector Gain	:+0.00 kV
Threshold	:1000

[MS Table]
--Group 1 - Event 1--

Start Time	:5.00min
End Time	:60.13min
ACQ Mode	:Scan
Event Time	:0.20sec
Scan Speed	:3333
Start m/z	:40.00
End m/z	:650.00

Sample Inlet Unit :GC

[MS Program]
Use MS Program :OFF
